# Supplementary material for: Spatial distribution and determinants of solitary childbirth in Ethiopia: Evidence from the 2019 interim demographic and health survey
Source: PLoS One. 2025 Sep 26;20(9):e0333603. doi: 10.1371/journal.pone.0333603 (PMC12469165; doi:10.1371/journal.pone.0333603)
Supplement: Attachment — Submitted filename: Peer Review Comments.docx [file pone.0333603.s002.docx]

**Spatial Distribution and Determinants of Solitary Child Birth in Ethiopia: A case of Interim Ethiopian Demographic and Health Survey 2019**

**Peer Review**

1. **Title**

**Title**: The topic: Spatial Distribution and Determinants of Solitary Child Birth in Ethiopia: A case of Interim Ethiopian Demographic and Health Survey 2019 can be refined

The topic can be refined as “**Spatial Distribution and Determinants of Solitary Childbirth in Ethiopia: Evidence from the 2019 Interim Demographic and Health Survey**”

This revision of the title above was guided by several considerations:

- Terminological Precision: The phrase *“Solitary Child Birth”* was corrected to *“Solitary Childbirth”* to reflect standard usage in academic literature. Additionally, if the term refers to births occurring without skilled attendants or outside health facilities, further refinement—such as *“Unattended Births”* or *“Home Deliveries”*—may be warranted to ensure conceptual clarity and international relevance.
- Improved Scholarly Tone: The phrase *“A case of”* was replaced with *“Evidence from”* to convey a more rigorous and empirical framing. “Evidence from” signals that the study draws on nationally representative data and supports analytical generalization, which is more appropriate for a peer-reviewed journal.
- Structural Coherence and Citation Norms: The reference to the data source was streamlined from *“Interim Ethiopian Demographic and Health Survey 2019”* to *“2019 Interim Demographic and Health Survey.”* This reordering improves readability and aligns with how DHS datasets are typically cited in global health and demographic research. The national context (Ethiopia) is already established earlier in the title, making repetition unnecessary.

Overall, the refined title maintains the original intent while enhancing its clarity, professionalism, and appeal to journal editors and reviewers. It positions the study as a robust, data-driven analysis of maternal health patterns in Ethiopia, grounded in spatial and demographic inquiry.

2. **Abstract – Suggestions for Improvement**

- The Authors should clarify the problem statement - Refine the opening sentence to more clearly establish the public health significance of solitary childbirth. For example, specify the burden or consequences in terms of maternal morbidity or mortality to strengthen the urgency.
- The Authors should improve flow and structure - Consider restructuring the abstract into clearly labelled segments (e.g., Background, Methods, Results, Conclusion) or ensure smooth transitions between them. This will enhance readability and help readers quickly grasp the study’s scope.
- The Authors should add specificity to the study methods - Briefly mention how solitary childbirth was defined or measured in the DHS dataset. This adds transparency and helps readers understand the operationalization of the key variable.
- The Authors should refine the conclusion for the study impact - Strengthen the final sentences by emphasizing actionable recommendations or policy relevance. For instance, suggest how hotspot identification could inform targeted maternal health interventions or ANC outreach programmes.

**3. Introduction**

- The Authors should clarify the conceptual definition of “solitary childbirth” early on, distinguishing it from unattended or home births if applicable.
- The Authors should strengthen the rationale by citing recent Ethiopian or regional studies that highlight the public health relevance of solitary births.
- The Authors should consider framing the issue within broader maternal health goals (e.g., SDG 3) to emphasize policy relevance.
- The Authors should streamline the background to focus more sharply on the gap this study addresses.

**4. Methods**

- The Authors should provide more detail on how solitary childbirth was operationalized in the dataset—what specific survey question or criteria were used?
- The Authors should clarify the sampling strategy and weighting procedures to enhance transparency and reproducibility.
- The Authors should justify the use of multilevel logistic regression by briefly explaining the hierarchical structure of the data.
- The Authors should include ethical considerations or approval details, especially since the study involves human subjects.

**5. Results Presentation**

- The Authors should use tables and maps to visually support spatial findings—especially hotspot regions.
- The Authors should ensure consistency in reporting confidence intervals and p-values across all results.
- The Authors should highlight key findings with brief interpretive comments to guide the reader.
- The Authors should consider stratifying results by region or demographic group to deepen insights.

**6. Discussion**

- The Authors should expand on why certain regions (e.g., Benishangul-Gumuz, SNNPR) may have higher rates of solitary birth—link to cultural, infrastructural, or policy factors.
- The Authors should compare findings with similar studies in sub-Saharan Africa to situate the results in a broader context.
- The Authors should address potential confounders or biases that may have influenced the associations.
- The Authors should avoid overgeneralization by acknowledging the limitations of cross-sectional data in establishing causality.

**7. Strengths and Limitations of the study**

- The Authors should emphasise on the strength: Use of nationally representative DHS data enhances generalizability.
- The Authors should highlight the strength: Integration of spatial and multilevel analysis provides a robust methodological approach.
- The Authors should underscore on the limitation: Potential recall bias in self-reported childbirth experiences.
- The Authors should accentuate on the Limitation: Lack of qualitative data limits understanding of women's lived experiences.

**8. Implications of Findings**

- The Authors should emphasize how identifying hotspot regions can inform targeted maternal health interventions.
- The Authors should suggest integrating community-based education and ANC outreach in pastoral regions.
- The Authors should highlight the role of policy in addressing structural barriers to assisted childbirth.
- The Authors should recommend collaboration with local health systems to improve birth preparedness and support networks.

**9. Conclusion/Recommendations**

- The Authors should reiterate the urgency of addressing solitary childbirth as a public health concern.
- The Authors should offer specific, actionable recommendations for health planners and policymakers.
- The Authors should consider proposing future research directions, such as qualitative studies or longitudinal tracking.
- The Authors should ensure the conclusion aligns with the study’s objectives and key findings.

**10. Proofreading and Editing**

- The Authors should correct some minor typographical errors and ensure consistent formatting.
- The Authors must improve sentence flow by reducing redundancy and tightening phrasing.
- The Authors should standardize terminology (e.g., “solitary childbirth” vs. “solitary birth”) throughout the manuscript.
- The Authors should ensure clarity in transitions between sections to enhance readability.

**9. References/In-Citations**

- The Authors should verify that all cited studies are current, relevant, and properly formatted according to journal guidelines.
- The Authors should include more regional or country-specific literature to strengthen contextual grounding.
- The Authors should ensure in-text citations match the reference list and are consistently styled.
- The Authors should consider citing WHO or UNFPA reports to support global maternal health framing.
